# Indiana State Dental Associaion

**Published:** 1864-08

**Authors:** Jos. Richardson, A. M. Moore

**Affiliations:** Indianapolis; Indianapolis


### INDIANA STATE DENTAL ASSOCIAION.

*Indianapolis, June 28th,* 1864.

The Indiana State Dental Association, in response to a call by the President, convened at the rooms of the "Ames Institute," Tuesday morning, June 28th, 1864. The meeting was called to order at 10 o'clock, the President, Dr. P. G. C. Hunt, in the chair. Members present: Drs. A. M. Moore, Lafayette, Ind.; J. F. Canine, Crawfordsville, Ind.; P. G. C. Hunt, Indianapolis, Ind.; J. F. Johnson, Indiannpolis, Ind.; G. A. Wells, Indianapolis, Ind.

On motion, the President appointed the following Committees:

*On Order of Business:*—Drs. A. M Moore, J. F. Johnson and J. F. Canine.

*On Membership:*—Drs. J. F. Johnson, G. A. Wells and J. F. Canine.

On motion adjourned until 2 o'clock, P. M.

AFTERNOON SESSION.

2 o'clock.

President in the chair.

The Committee on Order of Business made the following report, which was adopted.

1. Report of Committees.
2. Admission of Members.
3. Election of Officers.
4. President's Address.
5. Reading of Original Essays.
6. Discussion of following topics: 1. Treatment of Exposed Pulps. 2. Stopping Teeth. 3. Pivoting Teeth. 4. Mechanical Dentistry.
7. Miscellaneous Business.

DR. A. M. MOORE,<br>" J. F. JOHNSON, } *Committee.*<br>" J. F. CANINE.

The Committee on Applications for Membership made the following report, which was adopted; the candidates balloted for and elected.

"The Committee on Applications respectfully report the following gentlemen as applicants for membership: Drs. Jos. Richardson, D. M. Weld and Robert Van Valzah, of Terre Haute, Drs. C. C. Burgess, Merit Wells and A. E. Pursell, of Indianapolis.

DR. J. F. JOHNSON,<br>" G. A. WELLS, } *Committee.*<br>" J. F. CANINE,

The balloting for officers for the ensuing year, being next in order, the following gentlemen were elected:

*President*—Dr. A. M. MOORE.

1*st Vice-President*—Dr. J. F. CANINE.

2*d* " " " D. M. WELD.

3*d* " " " C. C. BURGESS.

*Rec. and Cor. Secretary*—Dr. J. RICHARDSON.

*Treasurer*—Dr. G. A. WELLS.

The retiring President then delivered an able and instructive address, which was well received, and a copy requested for publication.

The President elect, having been conducted to the chair, assumed its duties with some excellent and pertinent prefatory remarks.

The subject of the treatment of exposed nerves coming next in order, was discussed by most of the members present.

Adjourned until 9 o'clock, A. M., to-morrow.

SECOND DAY.—MORNING SESSION.

9 o'clock.

Meeting called to order, the President in the chair.

The subject of stopping teeth was taken up and discussed, all the members present participating.

On motion, the election of delegates to represent the Association at the next annual meeting of the American Dental Association at Niagara Falls, was made the special order of business for 3 o'clock this P. M.

Adjourned until 2 o'clock this afternoon.

AFTERNOON SESSION.

President in the chair. Meeting called to order at 2 o'clock.

A general discussion on the subject of pivoting teeth was had, eliciting from members present some novel modes of performing this operation.

The appointment of delegates to American Dental Association coming in order, resulted, by ballot, in the choice of Drs. P. G. C. Hunt and Jos. Richardson. The President was authorized to appoint substitutes in case of inability of those regularly elected to attend.

A consideration of the subject of mechanical Dentistry closed the discussion of subjects regularly reported.

On motion, adjourned to meet in the City of Indianapolis, on the last Tuesday in June, 1865.

DR. JOS. RICHARDSON, *Sec.*

DR. A. M. MOORE, *President.*

